# The Impact of a Modality Switch During Isokinetic Leg Extensions on Performance Fatigability and Neuromuscular Patterns of Response

**DOI:** 10.3390/s25134013

**Published:** 2025-06-27

**Authors:** John Paul V. Anders, Tyler J. Neltner, Robert W. Smith, Jocelyn E. Arnett, Richard J. Schmidt, Terry J. Housh

**Affiliations:** 1 The Human Performance Collaborative, Enterprise for Research, Innovation and Knowledge, The Ohio State University, Columbus, OH 43210, USA; 2 Health and Human Performance, College of Liberal Arts and Education, University of Wisconsin-Platteville, Platteville, WI 53818, USA; neltnert@uwplatt.edu; 3 Health, Human Performance and Sport, School of Science, Health, and Criminal Justice, Wayne State College, Wayne, NE 68787, USA; bosmith1@wsc.edu; 4 Exercise Physiology Laboratory, Department of Nutrition and Health Sciences, The University of Nebraska-Lincoln, Lincoln, NE 68588, USA; jarnett4@huskers.unl.edu (J.E.A.); rschmidt1@unl.edu (R.J.S.); thoush1@unl.edu (T.J.H.)

**Keywords:** performance fatigability, electromyography, isokinetic exercise, neuromuscular strategies

## Abstract

**Highlights:**

**What are the main findings?**

- Bilateral and unilateral muscle actions elicited similar neuromuscular activation strategies.
- Incorporating unilateral following bilateral muscle actions may potentiate greater metabolic stress.

**What is the implication of the main finding?**

- Practitioners should consider selecting exercises based on sport-specific needs.
- Following bilateral with unilateral muscle actions could be a method of eliciting greater muscular adaptations via metabolic stimulation of hypertrophic mechanisms.

**Abstract:**

Bilateral (BL) and unilateral (UL) muscle actions are commonly incorporated in training programs to achieve distinct goals, however, the mechanisms driving modality-specific training adaptations remain unclear. This study examined peak force, electromyographic (EMG) amplitude (AMP), and mean power frequency (MPF) of the non-dominant leg during isokinetic leg extensions performed as either a BL or BLUL combined modality. Twelve recreationally trained men (Mean ± SD; age = 20.8 ± 1.7 years; weight = 83.1 ± 15.7 kg; height = 178.2 ± 7.8 cm) attended 2 test visits that included BL and UL maximal isokinetic leg extensions at 180°·s^−1^ followed by a fatiguing task of either 50 BL or 25 BL followed immediately by 25 UL (BLUL) maximal, isokinetic leg extensions at 180°·s^−1^, in random order on separate days. The results demonstrated a 33.3% decline in peak force with a concomitant increase in EMG AMP across the fatiguing task, but there were no significant differences between conditions. For EMG MPF, the BLUL condition exhibited a 19.39% decline versus a 10.97% decline in the BL condition. Overall, the present study suggested there were no significant differences in neuromuscular activation strategies between the tested modalities. However, our findings indicated that incorporating UL muscle actions after a BL task may induce a greater degree of peripheral fatigue compared to sustained BL muscle actions. Practitioners might consider implementing UL exercises at the end of a training bout to induce greater metabolic stress.

## 1. Introduction

Performance fatigability is characterized as the decline in force production of activated muscles during exercise [1]. Previous research indicates that performance fatigability is task-specific, with studies reporting greater performance fatigability during unilateral (UL) compared to bilateral (BL) muscle actions in both submaximal and maximal exercise bouts [2,3,4]. While the mechanisms underlying these modality-specific differences remain unclear, it is hypothesized that the UL tasks pose a lower risk to whole-body homeostatic conditions compared to BL tasks due to UL tasks engaging less muscle mass [1]. As a result, BL tasks are subject to unique neuromuscular activation strategies, including interhemispheric inhibition (IHI) that reduces the cortical drive to the activated muscle and attenuates force production, which would not be experienced during UL tasks [5,6,7]. This would suggest that during fatigue, BL tasks may engage neuromuscular strategies that constrain force output as a protective mechanism against excessive fatigue. In contrast, UL tasks allow for maximal force expression, which may lead to a greater accumulation of fatigue. Our previous study demonstrated a greater performance fatigability during UL versus BL leg extensions, which was likely associated with a significant buildup of metabolic byproducts [2]. These findings align with the hypothesis that BL tasks are modulated by neural mechanisms that mitigate the development of peripheral fatigue.

Unilateral and BL muscle actions are commonly incorporated in training programs to achieve distinct, modality-specific adaptations. In general, BL training modalities have been prioritized for enhancing muscular strength and power, partly due to the greater absolute training loads that are lifted compared to UL modalities [8]. Unilateral training modalities are often employed for injury prevention and to develop sport-specific skills, such as kicking or change-in-direction drills, which primarily rely on UL movements [9]. Over time, long-term training studies have shown that strength and power improvements are greater when exercises are matched to the specific modality being trained [8,9]. The bilateral deficit, defined as the ratio between BL and UL force production, is often used to assess modality-specific differences in force production by homologous muscles [7]. Research indicates that UL training tends to promote a bilateral deficit (greater force during UL actions of both limbs than during BL actions) whereas BL training tends to promote bilateral facilitation (greater force during BL actions than the sum of UL forces from both limbs) [10,11]. However, the factors during acute training bouts that contribute to these modality-specific adaptations in force production remain unclear.

The differences in cortical drive between BL and UL tasks under acute training conditions [2,3,4], in addition to the plasticity of the bilateral deficit following chronic training [8,9], suggest that the modality-specific differences in training adaptations may be related to neuromuscular mechanisms. Under acute exercise conditions, BL tasks may involve neuromuscular modulation to limit force output and mitigate fatigue, whereas UL tasks permit greater force production and, consequently, greater fatigue accumulation. The purpose of the present study was to investigate whether switching from BL to UL muscle actions midway through the fatiguing task would lead to significant differences in performance fatigability and neuromuscular excitation strategies compared to sustained BL muscle actions. The inclusion of a BL to UL switch condition may identify differences in excitation strategies that otherwise could not be detectable if each modality were examined independently. Electromyography (EMG) was utilized in the present study to provide insights regarding peripheral (i.e., at the level of exercised muscle) neuromuscular excitation (EMG AMP) and muscle fiber conduction velocity (EMG MPF) [12]. Specifically, EMG AMP reflects changes in the magnitude of motor unit recruitment, firing rate, and synchronization, while EMG MPF reflects changes in muscle fiber conduction velocity in responses to changes in metabolic milieu during exercise [12,13]. Based on previous findings [2,3,4,14], we hypothesized that: (1) The inclusion of UL repetitions in the combined condition would result in a greater magnitude of performance fatigability compared to the BL-only condition; (2) The greater performance fatigability during the combined condition would be associated with significantly greater neuromuscular excitation; and (3) The greater performance fatigability would be associated with a greater peripheral fatigue, as indicated by changes in action potential conduction velocity.

## 2. Materials and Methods

### 2.1. Participants

Twelve recreationally trained men (Mean ± SD; age = 20.8 ± 1.7 years; weight = 83.1 ± 15.7 kg; height = 178.2 ± 7.8 cm) volunteer as participants for the present study. An a priori power analysis was conducted using G*Power (Version 3.1.9.6; Heinrich-Heine-Universität Düsseldorf, Düsseldorf, Germany) [15] to determine the minimum sample size necessary for detecting significant changes to peak force and EMG MPF. Based on our previous work [2], an anticipated effect size of Cohen’s d = 1.10 was used with an alpha level set to 0.05 and desired power of 0.80. The results indicated a minimum of 9 participants would be needed to detect mean differences in these neuromuscular parameters. Prior to participation, individuals interested in the study reviewed an informed consent document that outlined the requirements and expectations for the study. Once they signed the informed consent, the participants completed a Health History Questionnaire where the participants were screened for injuries that would influence or prevent their maximal performance. The study was performed in accordance with the ethical standards as established by the 1964 Declaration of Helsinki and was approved by the University Institutional Review Board for Human Subjects (IRB Approval #: 20210721148FB).

### 2.2. Familiarization Visit

The first visit to the laboratory was an orientation session to familiarize the participants with the exercise protocol they would be performing during the subsequent two test visits. During the familiarization, the participants completed submaximal and maximal, UL and BL, isokinetic leg extensions on a calibrated Cybex 6000 dynamometer (Cybex, Division of Lumex Inc., Ronkonkoma, NY, USA). Range of motion was determined by setting a stopper at the participants’ leg extension angle and 90 degrees into flexion to indicate a full repetition. Specifically, the participants began by performing 5 BL and 5 UL, submaximal, isokinetic leg extensions at 180°·s^−1^, separately, followed by 5 BL and 5 UL, maximal isokinetic leg extensions at 180°·s^−1^, separately. Then, the participants performed 5 maximal, BL isokinetic leg extensions immediately followed by 5 maximal, UL isokinetic leg extensions to reflect the modality switch in the combined condition. For the familiarization and subsequent testing visits, all UL leg extensions were performed with the non-dominant leg as determined by kicking preference. Previous studies have indicated little to no interlimb differences in peak force and fatigability [16,17] and based on our previous work [2] the nondominant limb may exhibit fatigue-induced changes in neuromuscular excitation strategies. At the end of the familiarization visit, the participants scheduled their testing visits with a minimum of 48 h between visits.

### 2.3. Test Visits

A total of 2 testing visits were completed for the present study. Based on previous work, the familiarization and 2 testing visits likely did not result in a learning effect for the fatiguing tasks that would affect neuromuscular excitation strategies [18,19]. Randomization for fatiguing task order between also reduced the potential for a learning effect across all sessions. All testing visits began with a standardized warmup that consisted of cycle ergometry (Ergomedic 828E, Monark, Varberg, Sweden) at 50 watts for 5 min. The participants were then seated on the Cybex 6000 where researchers aligned the dynamometer head with the axis of rotation of the knee joint and a seat belt was secured across the chest and hips of the participant to minimize any extraneous movement. During the leg extensions, participants were asked to hold onto the handlebars located on either side of the thigh during each repetition. The participants performed 2 sets of 2 repetitions of each submaximal BL and UL, isokinetic leg extensions at 180°·s^−1^. The participants were given a 2 min rest after the completion of the warmup and prior to the onset of the pre-fatiguing tasks. This protocol, 50 repetitions at 180°·s^−1^, is an established isokinetic protocol to examine fatigue, known as the Thorstensson test [20], and was used in the present study to provide greater context for previous work that have utilized the same protocol [2,21].

Upon completion of the warmup, the participants performed pre-fatigue maximal testing that consisted of 2 UL, maximal, isokinetic leg extensions at 180°·s^−1^. The participants then completed a fatiguing task that consisted of either 50 BL repetitions or 25 BL repetitions immediately followed by 25 UL repetitions of maximal, isokinetic leg extensions at 180°·s^−1^, in random order on separate days. All repetitions were performed continuously, without any rest allotted between repetitions; verbal encouragement was given to ensure continuous exercise was performed. Immediately following the fatiguing task, the participants again completed 2 UL, maximal, isokinetic leg extensions at 180°·s^−1^. A brief transition period (~10–15 s) was allotted to reconfigure the Cybex computer settings between the fatiguing and post-fatigue tests, in order to minimize the influence of recovery on post-fatigue measurements.

### 2.4. Electromyography and Force Acquisition

**Figure 1:**
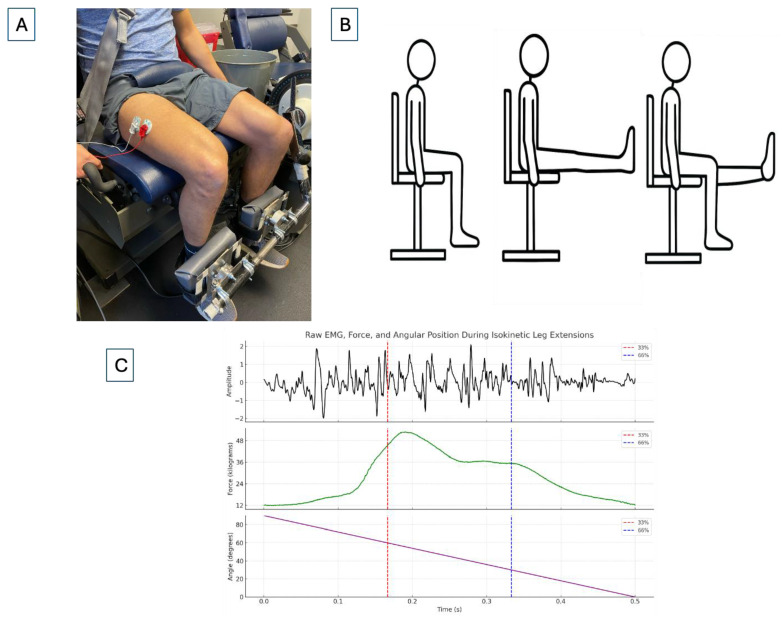
Example data acquisition during the fatiguing tasks including (**A**) electromyographic electrode and pancake loadcell placement, (**B**) starting and ending positions for the bilateral and unilateral leg extensions and (**C**) graphical example of the raw electromyographic, force, and angular position signals for a single repetition during the fatiguing task. Lines were placed at 33% and 66% of the full range of motion and indicate the section of the raw signal that was used for subsequent signal processing.

The electromyographic signal was assessed with a bipolar (30 mm center-to-center) surface EMG electrode arrangement (circular 4 mm diameter silver/silver chloride; Biopac Systems, Inc., Santa Barbara, CA, USA) for the vastus lateralis of the non-dominant limb (Figure 1). For each visit, the electrodes were placed 66% of the distance between the anterior superior iliac spine and the lateral portion of the patella and was orientated at an angle of 20° to approximate the pennation angle of the muscle fibers, with placement and preparation in accordance with SENIAM recommendations [22]. A reference electrode was placed over the anterior superior iliac spine. Prior to electrode placement, the location was shaved, abraded, and cleaned with an alcohol wipe to remove any excess skin cells or oils that might affect the fidelity of the EMG signal. Peak force data for the non-dominant leg was recorded from a pancake loadcell (Honeywell International Inc., Charlotte, NC, USA) located between the ankle of the participant and the lever arm of the isokinetic dynamometer and calibrated to report kilograms (kg) of force produced. This arrangement allowed investigators to quantify force production of the non-dominant leg only when performing bilateral muscle actions.

The raw EMG signal from the non-dominant vastus lateralis was sampled at 2000 Hz and was converted with a 12-bit analog-to-digital converter (Model MP 150; Biopac Systems, Inc., Goleta, CA, USA), amplified with a differential amplifier (gain: ×1000; EMG100C; Biopac Systems Inc., Goleta, CA, USA), and the digitized signal stored on a personal computer (G5 Dell Inc., Round Rock, TX, USA) for subsequent analyses. The EMG signal was bandpass filtered (forth-order Butterworth) at 10–500 Hz with all signal processing being completed with a customized program in LabVIEW programming software (Version 18.0f2; National Instruments, Austin, TX, USA). For each isokinetic repetition, the epoch for the EMG AMP and MPF was calculated from the middle 30° range of motion (between 30° and 60° with 0° indicating full flexion) to minimize the inclusion of data during the acceleration and deceleration phases of the muscle action [23]. The EMG AMP was then determined by root mean square analysis and EMG MPF was determined with fast Fourier transform. For each repetition during the fatiguing task, isokinetic peak force, EMG AMP, and EMG MPF were averaged across every 5 consecutive repetitions (i.e., average of repetitions 1–5 = repetition 5; average of repetitions 6–10 = repetition 10, etc.) to generate a total of 10 data points and every data point was normalized to the value at repetition 5.

### 2.5. Statistical Analysis

The Shapiro–Wilk Test was used to assess the normality of the data distribution for all variables. Separate ANCOVAs were conducted to evaluate differences in post-fatigue absolute values for peak force, EMG AMP, and EMG MPF in response to the fatiguing conditions (BL vs. BLUL), with pre-fatigue values included as covariates to control for baseline differences and to account for potential day-to-day variability in neuromuscular assessments. For normalized isokinetic peak force, EMG AMP, and EMG MPF values during the fatiguing task, the mean differences across the fatiguing task were examined with separate 2 (Condition: BL, BLUL) × 10 (Repetition: 5–50) repeated measures ANOVAs to examine the patterns of response. When appropriate, significant interactions and main effects were further examined with follow-up 1-way repeated measures ANOVAs and Bonferroni-corrected pairwise comparisons, respectively. For the repeated measures ANOVAs, a Greenhouse-Geisser correction was applied if Mauchly’s Test of Sphericity was significant. Effect sizes (partial eta squared, η^2^_p_ and 95% confidence intervals, 95%CI) were calculated for all ANOVAs and post hoc analyses, respectively, and an alpha of *p* < 0.05 was considered statistically significant for all analyses. All statistics were performed with IBM SPSS v. 28 (Armonk, NY, USA).

## 3. Results

### 3.1. Pre- and Post-Fatigue Neuromuscular Assessments

For isokinetic peak force, the adjusted pre-fatigue mean was 52.0 kg. The results of the ANCOVA indicated there were no significant differences between the BL (42.1 ± 8.79 kg) and the BLUL (42.2 ± 8.79 kg) conditions (*p* = 0.693, η^2^_p_ = 0.00). For EMG AMP, the adjusted pre-fatigue mean was 613.5 μV. The results of the ANCOVA indicated there were no significant differences between the BL (731.2 ± 248.6 μV) and BLUL (711.1 ± 248.6 μV.) conditions. For EMG MPF, the adjusted pre-fatigue mean was 76.3 Hz. The results of the ANCOVA indicated there were no significant differences between the BL (65.8 ± 16.7 Hz) and BLUL (67.7 ± 16.7 Hz) conditions.

### 3.2. Patterns of Response for Normalized Isokinetic Peak Force

**Figure 2:**
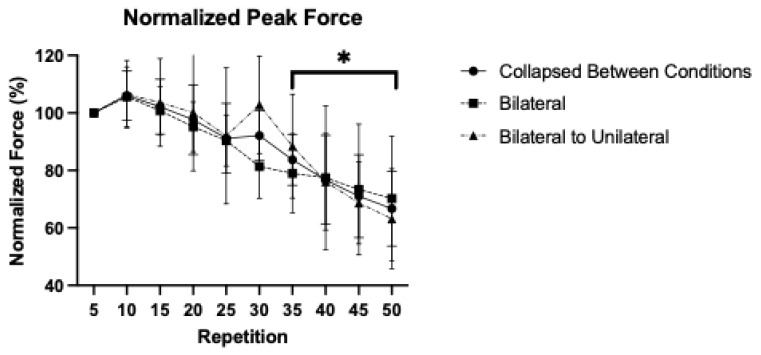
Mean (±SD) of the isokinetic peak force, for bilateral, bilateral to unilateral, and collapsed between conditions during maximal, isokinetic leg extensions. All values were normalized as a percentage of the mean at repetition 5. * Indicates a significant (*p* < 0.05) difference from the value at repetition 5 when collapsed between conditions.

For normalized isokinetic peak force, the 2 × 10 repeated measures ANOVA demonstrated no significant interaction (*p* = 0.869, η^2^_p_ = 0.171), but a significant main effect for Time (*p* = 0.041, η^2^_p_ = 0.195). The post hoc Bonferroni-corrected pairwise comparisons demonstrated that compared to the value at repetition 5 (100 ± 0.00%), repetitions 35 (83.47 ± 8.90%; *p* = 0.002; 95%CI = 5.06, 27.54), 40 (76.73 ± 15.49%; *p* = 0.013; 95%CI = 3.71, 42.83), 45 (71.03 ± 14.38%; *p* = 0.001; 95%CI = 10.82, 47.13), and 50 (66.71 ± 13.03; *p* < 0.001; 95%CI = 16.85, 49.74) were significantly lower (Figure 2).

### 3.3. Patterns of Response for Normalized Electromyographic Amplitude

**Figure 3:**
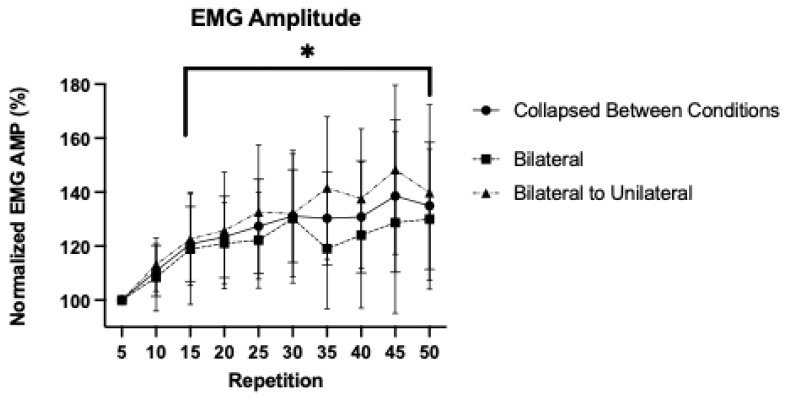
Mean (±SD) of the electromyographic amplitude (EMG AMP), for bilateral, bilateral to unilateral, and collapsed between conditions during maximal, isokinetic leg extensions. All values were normalized as a percentage of the mean at repetition 5. * Indicates a significant (*p* < 0.05) difference from the value at repetition 5 when collapsed between conditions.

For normalized EMG AMP, the 2 × 10 repeated measures ANOVA demonstrated no significant interaction (*p* = 0.077, η^2^_p_ = 0.181), but a significant main effect for Time (*p* < 0.001, η^2^_p_ = 0.533). The post hoc Bonferroni-corrected pairwise comparisons demonstrated that compared to the value at repetition 5 (100 ± 0.00%), repetitions 15 (120.81 ± 13.92%; *p* = 0.014; 95%CI = 3.24, 38.38), 20 (123.40 ± 15.08%; *p* = 0.010; 95%CI = 4.36, 42.43), 25 (127.42 ± 17.45; *p* = 0.009; 95%CI = 5.39, 49.45), 30 (131.17 ± 17.20; *p* = 0.003; 95%CI = 9.45, 52.189), 35 (130.30 ± 17.28%; *p* = 0.004; 95%CI = 8.48, 52.12), 40 (130.82 ± 20.17%; *p* = 0.014; 95%CI = 4.67, 56.97), 45 (138.53 ± 28.24%; *p* = 0.028; 95%CI = 2.87, 74.19), and 50 (134.93 ± 23.56%; *p* = 0.015; 95%CI = 5.19, 64.67) were significantly greater (Figure 3).

### 3.4. Patterns of Response for Normalized Electromyographic Mean Power Frequency

**Figure 4:**
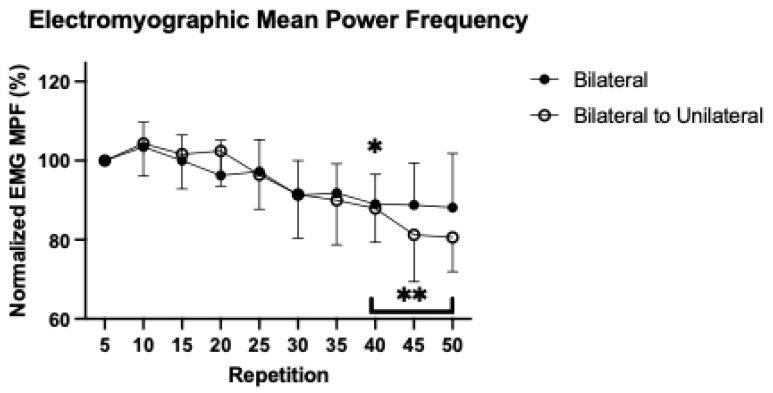
Mean (±SD) electromyographic mean power frequency (EMG MPF) for the bilateral (•) and bilateral to unilateral (ο) conditions during maximal, isokinetic leg extensions. All values were normalized as a percentage of the mean at repetition 5. * Indicates a significant (*p* < 0.05) difference from the value at repetition 5 for the bilateral condition. ** Indicates a significant (*p* < 0.05) difference from the value at repetition 5 for the bilateral to unilateral condition.

For normalized EMG MPF, the 2 × 10 repeated measures ANOVA demonstrated a significant Condition by Time interaction (*p* = 0.041, η^2^_p_ = 0.185; Figure 4). For the BL condition, there was a significant (*p* < 0.001; η^2^ = 0.440) follow-up repeated measures ANOVA, with Bonferroni-corrected pairwise comparisons which indicated that compared to repetition 5 (100 ± 0.00%), repetition 40 (89.03 ± 7.48%; *p* = 0.016; 95%CI = 1.32, 20.41) was significantly lower.

For the BLUL condition, there was a significant (*p* < 0.001; η^2^ = 0.703) follow-up repeated measures ANOVA, with Bonferroni-corrected pairwise comparisons which indicated that compared to repetition 5, repetitions 40 (78.90 ± 8.56%; *p* = 0.021; 95%CI = 1.29, 22.91), 45 (81.28 ± 11.86%; *p* = 0.009; 95%CI = 3.74, 33.70), and 50 (80.64 ± 8.68%; *p* < 0.001; 95%CI = 8.41, 30.33) were significantly lower (Figure 4).

## 4. Discussion

The purpose of the present study was to investigate whether BL and UL isokinetic muscle actions employ distinct neuromuscular excitation strategies under fatiguing conditions. It was hypothesized that switching from BL to UL leg extensions would increase force production and neuromuscular excitation in the working limb, ultimately leading to greater performance fatigability and notable differences in neuromuscular responses by the end of the fatiguing task. The results indicated that the combined BLUL condition did not produce significant differences in peak force or normalized EMG AMP compared to the BL-only condition. However, EMG MPF, a measure of peripheral fatigue, exhibited a greater decline in the combined condition compared to the BL-only condition. Overall, while both conditions elicited similar changes in peak force and neuromuscular excitation, the greater decline in EMG MPF during the switch condition suggested that incorporating UL muscle actions may potentiate the development of peripheral fatigue relative to BL-only exercises.

In the present study, both conditions elicited an approximate 33.3% decline in force during the fatiguing task (Figure 2). The implementation of a modality switch from BL to UL leg extensions allowed investigators to directly compare force output and neuromuscular excitation between BL and UL muscle actions during fatiguing leg extensions. While we have previously demonstrated that UL muscle actions typically elicit greater changes in performance fatigability likely due to greater peripheral fatigue [2], we were unable to directly examine whether these modality-specific differences were associated with changes in neuromuscular excitation during the fatiguing task. While no studies have compared BL and UL tasks during a modality switch, previous studies [2,3,4] have demonstrated that during lower body muscle actions, UL tasks elicit a greater decline in force production than BL tasks. For example, Rossman et al. [4] reported a 44 ± 6% decline in voluntary activation and approximately 25% decline in maximal voluntary force (MVC) following UL leg extensions to failure, compared to a 33 ± 7% and approximately 12% declines in involuntary activation and MVC, respectively, for the BL condition. The findings of the present study, however, were consistent with those of Koral et al. [14] who reported no differences in the patterns of responses for force during 1 min BL or UL maximal isometric knee extensions. The magnitude of fatigability observed during the BL task in the present study was comparable to that reported by Rossman et al. [4] for a UL task. Notably, the present study implemented a discrete protocol (50 repetitions) with a maximal modality, rather than a time-to-task failure approach with a submaximal modality [4]. Future studies utilizing a time-to-task failure protocol with an isokinetic task may elicit a greater decline in force production and more pronounced alterations in neuromuscular excitation strategies.

Thomas et al. [1] suggested that performance fatigability is modulated by the magnitude of muscle mass activated during the task that competes with other physiological systems (e.g., cardiovascular, respiratory) vital for whole-body homeostasis. As BL tasks engage a greater amount of muscle mass, they may undergo greater neuromuscular modulation by group III/IV afferent neurons to minimize perturbations to homeostatic conditions than UL tasks and therefore, may exhibit an attenuated performance fatigability [1]. In the present study, pre- and post-fatigue assessments were conducted to evaluate the residual effects of the fatiguing task on neuromuscular parameters and indicated that both conditions elicited comparable changes in peak force, EMG AMP, and EMG MPF. Additionally, the lack of difference in the patterns of response for peak force and EMG AMP during the fatiguing tasks (Figure 2 and Figure 3) may be due to the same modality at the onset of both conditions, eliciting a similar challenge to whole-body homeostatic conditions. Thus, neuromuscular modulation of group III/IV afferent neurons may have been stimulated to a similar extent [24,25] and resulted in parallel patterns of response during the fatiguing task between both conditions, regardless of the switch to UL muscle actions in the combined condition. While the present study utilized a discrete task completion (50 repetitions), it remains unclear whether a transition from BL to UL would attenuate afferent feedback from the disengaged limb, resulting in a longer time-to-task failure and a greater volume of work being performed than a sustained BL condition.

In the present study, the participants exhibited a similar initial increase in EMG AMP for both conditions that was sustained for the duration of the fatiguing task (Figure 3). In general, these findings were consistent with previous work demonstrating an initial increase in neuromuscular excitation that is sustained for the duration of the fatiguing task during near-maximal and maximal, dynamic leg extensions [2,26,27]. Previous research has suggested that BL and UL modalities may demonstrate distinct excitation patterns as the result of unique cortical activation strategies, such as IHI, which is characterized by a higher magnitude of neural drive during UL compared to BL muscle actions [5,6,7,28]. The methodology in the present study was unable to demonstrate that a switch from BL to UL muscle actions elicited significant changes in neuromuscular excitation during the fatiguing task or during the follow-up assessments. These findings were similar to Koral et al. [14] who reported no difference in force production during 1 min of BL or UL isometric leg extensions, and no significant differences between conditions for EMG AMP or voluntary activation levels in follow-up assessments. The present study utilized zero-mean and RMS processing of the EMG signal in the time domain to characterize neuromuscular excitation. Alternative approaches, such as zero-crossing rate analysis [29] may offer more detailed and distinct methods for quantifying fatigue-related changes in the time-domain characteristics of the EMG signal. Overall, the findings indicated that both modalities elicited similar neuromuscular excitation responses during the fatiguing tasks, suggesting that distinct cortical activation strategies may not translate to measurable differences in neuromuscular excitation or force production during, and in response to, a mixed-modality condition.

The decline in force without a change in neuromuscular excitation, a characteristic of excitation–contraction coupling failure [30], suggested that the performance fatigability in the present was driven by peripheral rather than central mechanisms. The MPF is a spectral measure of EMG that has been used as a proxy measure for peripheral fatigue [31,32,33]. The development of peripheral fatigue has been associated with changes in the metabolic milieu within the muscle, including a buildup of calcium, inorganic phosphate, and hydrogen ions that have been hypothesized to affect calcium dynamics in the sarcoplasmic reticulum [30]. Declines in EMG MPF have been attributed to slower muscle fiber conduction velocities that change in response to the buildup of metabolic byproducts [32,34,35,36,37]. Previous work has demonstrated that the action potential conduction velocity of the knee extensors decreases in response to fatiguing exercise and has been attributed to changes in the metabolic milieu within the muscle [38]. The maximal nature of isokinetic muscle actions likely recruited all muscle fibers of the VL, including type II fibers that are larger [39], and thus have a greater action potential conduction velocity [40]. Furthermore, some evidence exists that type II fibers may be located more superficially of the VL [41]. Thus, while muscle excitation remained consistent through the duration of the fatiguing tasks, the decline in EMG MPF suggested that the participants likely experienced significant metabolic changes within the muscle that impaired muscle contractility. The robust change in EMG MPF during the fatiguing task may be attributed to the fatigability of high-threshold, Type II fibers in the VL while providing limited context for type I fiber neuromuscular strategies. Ultimately, these findings suggested that performance fatigability was primarily driven by excitation–contraction coupling failure and peripheral mechanisms, rather than central mechanisms.

The greater reduction in EMG MPF during the combined condition suggested that the transition from the BL to the UL modality may have accelerated the buildup of metabolic byproducts beyond that exhibited during the BL-only condition. This is consistent with previous work that has characterized a greater decline in EMG MPF for UL compared to BL muscle actions [2]. The present study extends these findings and suggests that the inclusion of a UL modality following a BL task may potentiate a greater decline in EMG MPF than sustained BL-only muscle actions and may be attributed to a greater metabolic buildup. Practitioners have utilized unique training paradigms, such as blood flow restriction, to elicit significant metabolic loads that stimulate hypertrophic adaptations in skeletal muscle [42,43,44]. The findings of the present study suggested that the inclusion of UL movements, either independently or as a compound set to BL muscle actions, may be implemented to elicit greater metabolic stimuli. It remains unclear whether the regular assessment of spectral changes in the EMG signal can be used to indicate metabolic stimulation that may stimulate hypertrophic mechanisms. Future research should examine whether the greater performance fatigability elicited by UL muscle actions under acute training sessions precipitate greater skeletal muscle adaptations across a training cycle.

The present study offers valuable insights into the peripheral manifestations of neuromuscular function during fatiguing muscle contractions. This study utilized surface EMG to examine neuromuscular excitation strategies under fatiguing conditions, and thus its findings should be interpreted considering known methodological limitations. These limitations include the risk of cross-talk from adjacent quadriceps muscles (e.g., vastus medialis, rectus femoris) that may have contaminated the EMG signal during the fatiguing task [45]. During dynamic exercise, and under fatiguing conditions, the EMG signal becomes non-stationary and may compromise the spectral interpretation, particularly when analyzed with Fourier transformations [46]. The EMG signal also does not follow a linear relationship with force production and thus should not be considered a proxy for neural drive or muscle excitation [46]. Furthermore, the use of surface EMG limited our ability to directly assess changes in central mechanisms of fatigue, as cortical drive and central nervous system activity were not directly measured. Previous research suggested that BL and UL modalities may involve distinct cortical activation strategies, which were not explored in this study [7]. Additionally, the present study only included male participants, leaving it unclear whether similar neuromuscular responses to fatigue would be observed in females. Future studies should aim to incorporate measures of central fatigue and include both male and female participants to provide a more comprehensive understanding of neuromuscular function during fatiguing tasks.

## 5. Conclusions

In conclusion, the present study suggested there were no significant differences in neuromuscular activation strategies between the tested modalities. However, our findings indicated that incorporating UL muscle actions after a BL task may induce a greater degree of peripheral fatigue compared to sustained BL muscle actions. Overall, practitioners might consider implementing UL exercises at the end of a training session to induce greater metabolic stress. Future research is necessary to investigate whether the greater peripheral fatigue observed when UL exercises were incorporated (as indicated by EMG MPF) translates into superior muscular adaptations compared to BL-only exercises.

## Data Availability

The raw data supporting the conclusions of this article will be made available by authors on request.

## Abbreviations

The following abbreviations are used in this manuscript:

95% CI	95% Confidence Interval
AMP	Amplitude
ANOVA	Analysis of Variance
ANCOVA	Analysis of Covariance
BL	Bilateral
EMG	Electromyography
IHI	Interhemispheric Inhibition
MPF	Mean Power Frequency
MVC	Maximum Voluntary Contraction
UL	Unilateral
